# Letter from Philadelphia

**Published:** 1879-05

**Authors:** 


### ARTICLE XIV.

### LETTER FROM PHILADELPHIA.

*Messrs. Editors:* — Medical organizations were never in a more favorable and flourishing condition in Philadelphia than they are at present. In addition to our County Medical Society, which was organized in 1848, and recently incorporated (1877), we have the Pathological Society, the Obstetrical Society, the College of Physicians, all well attended, and there are some evidences of a new organization, which shall be devoted entirely to surgery. Not long ago a resolution was passed by the County Medical Society, authorizing the formation of sections by its members, for the consideration and cultivation of especial branches; that is to say, if a dozen members interested in pathology, microscopy, laryngology, or any kindred topic, were to apply to the Society for the establishment of a section on pathology, for instance, to be called the Pathological Section of the Philadelphia County Medical Society, permission would be granted, with the understanding that it should involve no expense to the Society. Whether the surgeons who are moving in the matter will avail themselves of this or not is yet to be determined. The College of Physicians owns one of the finest medical libraries in America, deposited in a fire-proof building, in which there are several fine rooms for holding meetings, and it is in this place that the previously named societies assemble. In addition to these, there are a number of smaller medical societies, for the convenience of physicians in distant parts of the city, who are unable to attend the County Medical Society; some of them are incorporated, like the West Philadelphia Book Club, which meets regularly as a medical society, but others are simply small medical associations, existing in almost all the outlying sections of the city, and which should be really branches of the County Medical, as their objects and methods are identical. If we include scientific associations, which are largely made up of physicians, like our Academy of Natural Sciences, with its sections upon biology, and microscopy, anthropology and comparative anatomy, and other topics cognate to medical science, it may be inferred, and correctly, that our physicians are intellectually active, and socially harmonious. It is true that the profession in Philadelphia is annoyed to a less degree by irregulars and charlatans than perhaps any other large city. One reason for this, and probably one that has acted more directly than any other to bring about this desirable state of affairs, is the fact that quacks are systematically ignored, both professionally and socially. They are treated with uniform politeness, but kept at a distance, so that no discussion or dispute may give them the opportunity of claiming popular sympathy by raising the cry of persecution. We have a board of censors to report upon all applicants for admission to the County Medical Society, but even their recommendation will not secure a man's election at the balloting. To this board are also referred all charges against members for unprofessional conduct, but no member has yet been excluded from the Society simply because he has adopted any exclusive practice ; in other words, he is not hustled out because he is a homœopath, but he generally finds that the atmosphere is less congenial, and either sends in his resignation or is dropped for non-payment of dues. In this way he is deprived of a gratuitous advertisement in the daily papers as a first-water martyr, and instead of winning popular sympathy he has no notice taken of him whatever, and he retires into obscurity. Homœopathy is drawing its last breath with us, and it is now nearly time for a new " ism " or " pathy." We should really not be surprised some morning to hear, with a great flourish of trumpets, that a brand-new medical practice has come to town, known as "electricism," or something similar, that cures all diseases without medicine, not by smelling the bottle, as recommended by Hahnemann, but by the searching and eliminating influence, say, of mystic magnetism. All other practice, of course, will at once become effete, and be stigmatized as old, or allopathic, or some other equally vague and correspondingly dreadful term.

To turn to a more congenial topic, we may refer briefly to the prospects of higher medical education. The University of Pennsylvania, having secured a moderate endowment for this purpose, has adopted a three years' graded course, and hopes soon to establish a preliminary examination. This movement has received the endorsement of the State Medical Society, but has not met the response it deserved from the profession; although there is not the slightest doubt of its ultimate success. The Jefferson College, having a very conservative management, still clings to the old methods of lecturing, and although recommending a three years' course, it is not made an essential prerequisite for graduation. The faculty, probably without exception, are in favor of adopting a graded course, and extending it to at least three years, Prof. Gross is full of the idea, as will be seen by his action at the Convention of Medical Colleges in Atlanta.

The compliment of a public dinner, which was given to Prof. Gross by his surgical friends, at the St. George hotel, Philadelphia, April 10, 1879, in commemoration of his completing his fifty-first year in the profession, was largely attended by prominent surgeons from all parts of the country, and was a grand success.

In regard to practice and operations there is much that is worthy of note: Dr. John H. Brinton operated upon two cases of stone in the bladder by the lateral operation, recently, at the clinic of the Jefferson Medical College Hospital. One patient, a boy of nine years with a 3¼ ounce urate calculus, made a good recovery; the other, a man of 56 years, died on the third day from exhaustion. There was some difficulty in extracting the stone in the latter case, as it was quite large, though soft and phosphatic, and had to be removed in fragments. It weighed four ounces and six drachms, and the operation was therefore unusually prolonged. In a case of a stone weighing over five ounces at the Woman's Hospital, colpo-cystotomy was recently performed, and the calculus perforated with a drill run by Bonwitt's dental engine, the stone was then easily crushed and removed. This patient died shortly afterward of abcess of the kindeys, but it was stated that the bladder, at the post mortem, was found to be in good condition.

Dr. John B. Roberts recently read a paper on paracentesis of the pericardium before the County Medical Society, and stated that he had collected nearly fifty cases, and the percentage of recoveries was nearly 47 per cent. He considers the operation perfectly justifiable, and advocates the use of Fitch's dome-trocar, with the aspirator, the fifth intercostal space being selected at a point one and a half inches to the left of the line of the mid sternum.

Hot Water as a Parturifacient, was the title of a paper read at the same meeting by Dr. Albert H. Smith, who has had large experience, and states that hot water injections should be used in every case of obstetrics. In the first stage of labor, it stimulates the uterus, and facilitates delivery; after parturition, it cleanses the canal and vagina, causes prompt, firm and persistent uterine contraction, and is the best agent for checking post-partum hæmorrhage known, used at a temperature of 46° to 49°, and thrown *not into the uterus, but against the cervix*, with a Davidson syringe, or a tube and funnel. After delivery, he had found hot water injections to completely relieve after-pains. He had also used it in ordinary menorrhagia with great satisfaction, and in cases of labor when he had reason to anticipate flooding, he was in the habit of using it in the first stage as a preventive, and with astonishing success.

The State legislature has again failed to pass the bill authorizing the formation of a State Board of Health.

With the exception of an epidemic of influenza in February and March, the city has been unusually and distressingly healthy for nearly a year; during 1878, there was not a single death from small-pox in Philadelphia. W.

PHILADELPHIA, April, 1879.

